# Cystic Fibrosis: Fighting Together Against Coronavirus Infection

**DOI:** 10.3389/fmed.2020.00307

**Published:** 2020-06-09

**Authors:** Sara Manti, Giuseppe Fabio Parisi, Maria Papale, Enza Mulè, Donatella Aloisio, Novella Rotolo, Salvatore Leonardi

**Affiliations:** Pediatric Respiratory Unit, Department of Clinical and Experimental Medicine, University of Catania, Catania, Italy

**Keywords:** cystic fibrosis, COVID 19, coronavirus, SARS-CoV-2, recommandation

## Introduction

In a critical moment like this in which all world population is hard fighting with limited weapons against coronavirus (COVID-19) infection, we strongly feel the duty and the need to provide real help and clear information to people with underlying health conditions, such as cystic fibrosis (CF), to protect themselves and ones living with patients with CF as best as possible.

**Figure 1:**
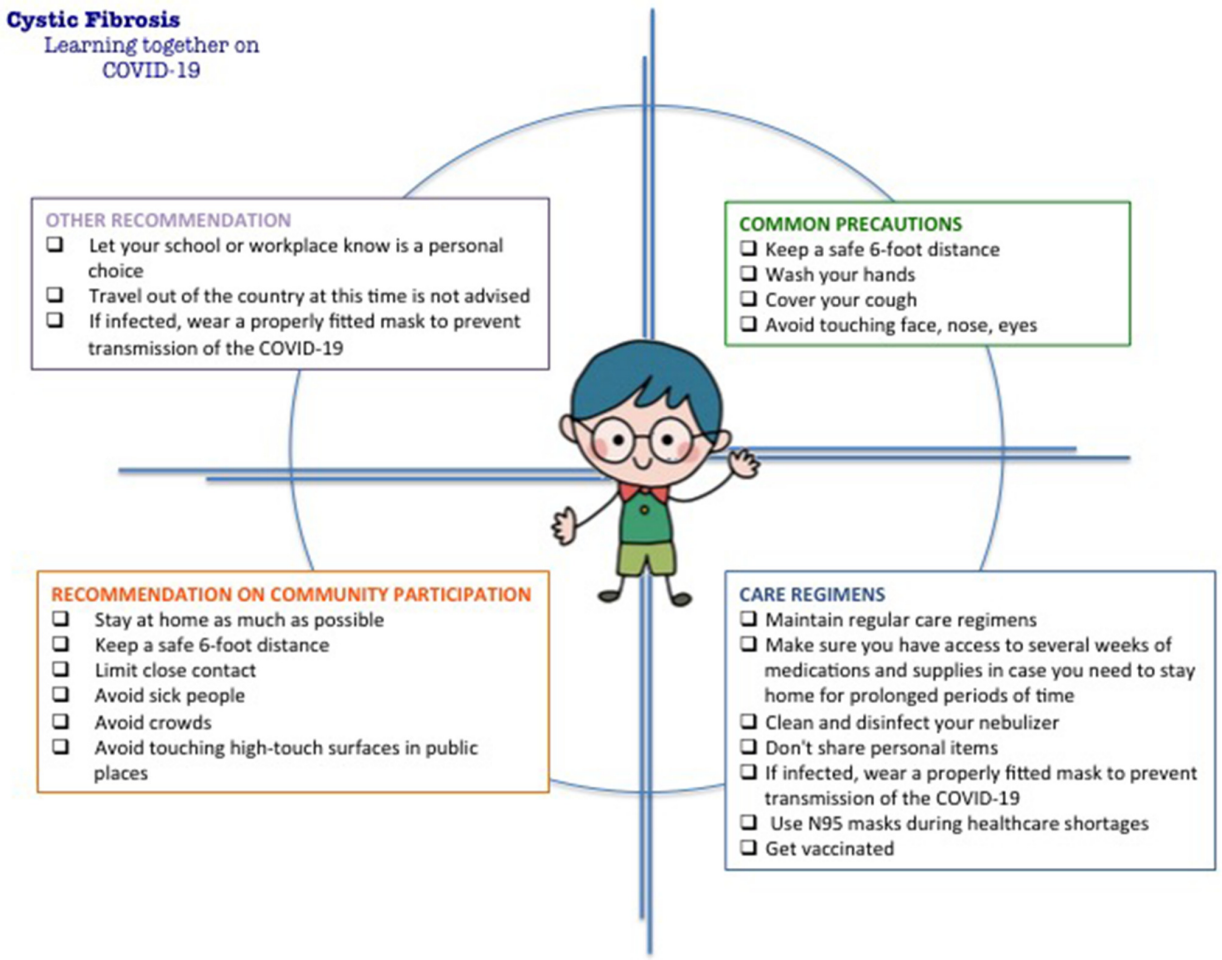
Suggested recommendations regarding the coronavirus (COVID-19) infection for patients at higher risk of severe illness.

To achieve this goal, we briefly summarized the current state of knowledge on COVID-19 infection in patients affected by CF. Moreover, we provided a simple flow-chart to summarize the recommendations suggested for patients at higher risk of severe illness, such as people affected by CF (Figure 1).

## Cystic Fibrosis and Viral Infections

Respiratory viral infections are common events throughout human life; however, when they occur in patients with chronic and/or underlying health conditions, their impact can become dramatic (1).

Among people with CF, respiratory viruses are associated with prolonged respiratory illness and show a clear association with pulmonary exacerbations, lung function decline, and risk of death (1, 2). Although the impact of respiratory viral infections on CF lung disease history is poorly understood, several mechanisms have been hypothesized to play a crucial role (3). The inflammation that characterizes the lower respiratory tract in CF is not primarily started by the genetic defect rather than viral infections that, already present in almost 40% of infants with CF at 3 months of age, impair the specific anti-bacterial defense, increase the adherence of bacteria to the mucous membrane, impact negatively nitric oxide synthase 2 (NOS2) activity, and, enhancing the pro-inflammatory cytokine production [interleukin (IL)-6 and IL-8], affect the immune defense in the human airway, resulting in severe respiratory illness in CF patients (4).

Specifically, CFTR deficiency results in changes in osmotic pressures and electro-neutrality which cause excessive sodium and water absorption, dehydration of the airway surface liquid and mucus layer as well as changing in pH airway surface liquid, favoring chronic retention of pathogens and a secondary inflammatory response (5). The increase in vesicular pH of cells with CF transmembrane conductance regulator (CFTR) deficiency seems to cause the inhibition of acid ceramidase resulting in the accumulation of lung epithelial ceramide that, in turn, increases cell death, stimulates bacterial binding to extracellular DNA, and initiate IL-1ß and chemokine synthesis (6). Moreover, the extensive plugging of the small airways by purulent mucus leads to a decrease in oxygen tension, which, in turn, can affect the host anti-bacterial defenses and favor bacterial growth (7). Another impaired mechanism described in cells of patients with CF is an abnormally high arachidonic acid to docosahexaenoic acid (AA/DHA) ratio which is associated with an increased inflammatory response (8).

The onset and persistence of inflammation in CF are critically important in host-pathogen interactions. Whether inflammation follows or rather precedes infection is still under debate but, undoubtedly, the inflammation of the airways is one of the key elements of the pathogenesis of the infections in CF patients. As a result of chronic inflammation, immune cells show multiple defects: neutrophils do not transport halide in the phagolysosome, thus, they are not efficient into oxidative killing (9); macrophages show a delayed phagolysosomal fusion and bacterial clearance as well as an enhanced toll-like receptor (TLR)4-dependent response to lipopolysaccharides (LPS) (10); naïve T lymphocytes are predisposed to differentiate toward a T helper (Th)17 phenotype (11). In parallel to all the above-mentioned mechanisms, the host factors also allow an increased virus replication. An impaired activation both of signal transducer and activator of transcription 1 (STAT 1) and NOS2, fundamental components of interferon (IFN)-y-mediated antiviral defense, increases the virus load and supports the severity of virus disease in CF (12). On the other hand, an exaggerated activation of the transcriptional regulatory complex nuclear factor (NF)-κB results in an increased production of proinflammatory cytokines (13). Therefore, it appears as in the context of the severity of pre-existing pulmonary and comorbidity CF disease, a lesser antiviral and greater inflammatory response are likely to contribute to severe respiratory illnesses of CF patients with viral infections, inducing and/or precipitating CF exacerbations (14).

Whether, historically, bacteria have been the predominant causes for respiratory exacerbations, these findings highlight as also viral agents can lead to clinical deterioration and, subsequently, morbidity, and mortality. CF pulmonary exacerbation rate is associated with the *Influenza A* and *B* viruses, *Respiratory Syncytial Virus* (RSV), *Parainfluenza virus types 1 to 4, Rhinovirus, Metapneumovirus*, and *Adenovirus* (15–18).

## Covid-19 and Cystic Fibrosis

Unlike human rhinovirus, consistently the most common respiratory virus affecting patients with CF, coronavirus (CoV) is an uncommon viral agent in this population (19–21). Moreover, in line with these findings, authors revealed that human CoV (HCoV) seem to have comparably little impact both on the rate of respiratory exacerbations and course of CF, with the exception for NL63 (1, 4). In this regard, Authors reported that HCoV seemed to have the same clinical impact of human *Rhinovirus* (HRV) in children with CF (20). Epidemiological findings reveal that HCoV is the second most prevalent respiratory virus in a 6-month winter period after HRV, and, these data are comparable to a cohort of age-matched healthy children (21). Moreover, in a study performed during a whole year period also including the summer season, Authors detected HCoV in a percentage of 0.8%, suggesting the marginal role of this virus in patients with CF (21).

Unfortunately, a new member of the large family of CoV, CoVID-19, is causing significant concern worldwide. Given the “young age” of the infection, specific literature data on CF patients are still not available but evidence has clearly assessed that people with underlying health conditions, including CF, seem to be a major risk of COVID-19-mediated serious illness. Several possible explanations for the severe clinical impact of SARS-CoV-2 virus in CF patients have been hypothesized. Similarly to HCoV-NL63, SARS-CoV-2 virus uses the angiotensin-converting enzyme-2 (ACE-2) as the main pathway for attachment and entry in the cell. Generally, following infection, a critical reduction in ACE-2 expression on the cell surfaces occurs; however, the rate of this down-regulation appears lower in CF patients when compared to patients not affected by CF (22). ACE-2 down-regulation is associated with an increase in the inflammatory response against the virus, thus, this event may be one of the determinants of severity of COVID-19 in CF patients (22). Moreover, genetic polymorphisms associated with an increase in ACE-2 expression have been related to a worse lung disease in CF patients (23). Lastly, the impact of COVID-19 is also associated with the baseline lung function of the CF patient, therefore, it is possible that subjects with severe lung disease are at higher risk to present an exacerbation, and, consequently, are more likely to develop severe COVID-19 form (24). Currently, to the best of our knowledge, COVID-19 has been confirmed in 58 patients with CF (age range, 6–28 years) (25). Ten of them were notified in Italy, and three of whom have been hospitalized. All infected patients were living in the endemic area, Lombardia, and acquired the infection from family members. Five patients with CF have been reported to have SARS-CoV-2 infection in Germany. Three patients with CF were also notified in Spain and one of them was transplanted (26). More detailed demographic and clinical findings of 40 out of 58 patients were collected only by eight countries including Australia, Canada, France, Ireland, Netherlands, New Zealand, United Kingdom (UK), and United States (US) (27). Of the 40 cases, 31 (78%) were symptomatic for SARS-CoV-2 at presentation, with 24 (60%) having a fever. The median age was 33 years (age range, 15–59 years). Thirty-eight percentage had CF-related diabetes mellitus (CFRD) and 70% were reported to have chronic bacterial pulmonary infection, of which 71% included *Pseudomonas aeruginosa*. One patient was pregnant and she was recovered, delivering a healthy baby. Eleven patients have been from post-lung transplant patients, who were on average 6 years post their transplant. Twenty-five (63%) patients were treated with new antibiotics: 10 subjects with oral antibiotics and 17 patients intravenous (IV) antibiotic treatment. Two people were receiving both oral and IV antibiotics. Fourteen people were reported as using CFTR modulators, 13 (33%) patients required oxygen, and only 1 out of 40 patients required invasive ventilatory support. Four out of 40 patients were admitted to Intensive Care Unit (ICU), and 3 of all them required oxygen. Twenty-eight out of 40 cases have been reported as clinically recovered from SARS-CoV-2, and no deaths were reported (27). No specific data are available about the incidence and outcomes within the pediatric CF population, and only one child affected by COVID-19 has been notified. A case of COVID-19 in a 1-month-old infant with CF has been reported; the patient presented with asymptomatic infection, despite his underlying condition (28). In summary, the above-mentioned findings show good recovery from COVID-19 even if in a heterogeneous CF cohort. Apparently, the disease course does not seem to differ from the general population, but the available epidemiological data are too small to draw conclusions. Dry cough, malaise, and fever are quite distinct from the symptoms of CF, thus, it is reasonable to hypothesize that patients with CF are recognizable, on the other hand, we cannot exclude that mild forms of COVID-19 may be mistaken for the common spectrum of CF symptoms. Obviously, taking into account the sparse available evidence, any conclusion can be reached about the incidence of COVID-19 both in adult and children with CF, and further and collaborative studies are required for a complete understanding of SARS- CoV2 infection impact on patients with CF. Moreover, people affected by CF should continue to strictly follow public health advice to protect themselves from COVID-19.

Although early and partial, these findings are encouraging and supporting the good job done to avoid SARS-CoV-19 infection. Compared to SARS-CoV-2 infection, H1N1 virus caused significant morbidity in patients with CF and resulting in respiratory deterioration, mechanical ventilation, and even death (29). At present, it is not possible to identify factors that might be protective, for example, use of long-term antibiotic therapy such as azithromycin, minimizing social contacts and self-isolation, cancellation of routine clinic appointments and procedures (respiratory function testing and bronchoscopy) to prevent unnecessary hospital visits and viral spread, and self-monitoring.

## Discussion

In absence of specific recommendations, we strongly encourage patients with CF to refer to the Centers for Disease Control and Prevention (CDC) guideline for people at higher risk for severe illness, defined as “older adults and people who have severe chronic medical conditions (e.g., heart, lung, or kidney diseases)”^1^. Additionally, individuals with CF and FEV1 <30% predicted with a predicted median survival longer than 5 years should not have a lower priority for intensive care (30).

To slow the spread and reduce the impact of the disease the following actions are recommended: (i) stay at home as much as possible; (ii) make sure to have access to medications and supplies at home for prolonged periods of time; (iii) take everyday precautions including keeping a safe 6-foot distance, limit close contact, avoid sick people and avoid crowds; wash hands often, avoid touching face, nose, eyes, avoid touching high-touch surfaces in public places; cover cough; clean and disinfect the nebulizer; get vaccinations.

### People With CF Should Maintain Their Regular Care Regimens

Let school or workplace is a personal decision. Travel out of the country at this time is not advised. Commonly available surgical and cloth masks have not been shown to protect against COVID-19; however, people who have or are likely to have SARS-CoV-2 infection will need to wear a mask to help control the spread of the virus to others. Moreover, wearing a properly fitted facemask (surgical or non-medical) it is also recommended when a healthy person leaves home, especially if they will be in contact with other people (30). All these recommendations are summarized in Figure 1.

In summary, we strongly believe that few, simple, and banal actions can be of great help and support to countering the difficult ongoing situation. Moreover, in the context of the COVID-19 pandemic, a transition from face-to-face clinics to multidisciplinary telemedicine care team could further protect CF patient from the risk of SARS-CoV-2 infection, preserving the CF care model.

## Author Contributions

SL developed the original idea and the final revision. SM, GP, MP, EM, DA, and NR wrote the manuscript. SM and GP revised firstly the manuscript and contributed to English revision and references update. MP, EM, DA, and NR made the final analysis and critical revision of the manuscript. All authors read and approved the final manuscript.

## Conflict of Interest

The authors declare that the research was conducted in the absence of any commercial or financial relationships that could be construed as a potential conflict of interest.

## Footnotes

^1^https://www.ecdc.europa.eu/sites/default/files/documents/RRA-seventh-update-Outbreak-of-coronavirus-disease-COVID-19.pdf
